# Discontinuation of biologic therapy in severe asthma: Evidence and strategies for safe withdrawal: A scoping review^☆^

**DOI:** 10.1016/j.waojou.2025.101107

**Published:** 2025-08-22

**Authors:** Johanna Ramirez-Villamizar, Ciro D. Ibarra-Enríquez, Juan Sebastián Galindo-Sánchez, Carlos Serrano-Reyes, Liliana Fernández-Trujillo

**Affiliations:** a Universidad Icesi, Facultad de Ciencias de la Salud, Calle 18 No. 122–135, Cali, 760031, Colombia; b Fundación Valle del Lili, Centro de Investigaciones Clínicas, Carrera 98 No. 18–49, Cali, 760032, Colombia; c Fundación Valle del Lili, Department of Internal Medicine, Pulmonology Service, Carrera 98 No. 18 - 49, Cali, 760032, Colombia; d Fundación Valle del Lili, Department of Internal Medicine, Allergy Service, Carrera 98 No. 18 - 49, Cali, 760032, Colombia

**Keywords:** Severe asthma, Biologic therapy, Discontinuation, Withdrawal, Scoping review

## Abstract

**Introduction:**

Severe asthma is characterized by poor disease control despite the use of high-dose inhaled corticosteroids and long-acting bronchodilators. Biologic therapies have revolutionized its management, allowing some patients to achieve remission. However, uncertainty remains regarding the optimal duration of treatment and the safest strategies for discontinuation. This study reviews the available evidence on the withdrawal of biologic therapy in patients with severe asthma in remission, evaluating their clinical outcomes.

**Methods:**

A literature review was conducted in PubMed, EMBASE, Epistemonikos, and LILACS up to May 2024, using terms related to severe asthma and discontinuation of biologic therapies. Studies evaluating asthma control after dose reduction or withdrawal of biologic treatment were included, considering outcomes such as exacerbations, lung function, and inflammatory biomarkers.

**Results:**

Of the 2494 studies identified, 23 articles were included after full-text review. Discontinuation of tezepelumab led to a gradual loss of clinical control in most patients, although baseline levels of inflammation were not reached. Regarding mepolizumab, 59% of patients experienced at least 1 significant exacerbation within the first year after withdrawal, suggesting the need for prolonged use. For omalizumab, results were heterogeneous: 67% of patients who continued treatment remained exacerbation-free, compared to 47.7 of those who discontinued it. Studies that implemented gradual tapering strategies showed higher success rates in discontinuation without loss of clinical control.

**Conclusion:**

Evidence suggests that discontinuation of biologic therapy should be individualized, and a minimum treatment duration of 5 years may be appropriate before considering withdrawal. Optimal candidates include those with sustained clinical control, stable lung function, suppressed inflammatory biomarkers, and no need for oral corticosteroids. Gradual decreasing strategies may optimize treatment withdrawal while minimizing the risk of relapse.

## Introduction

Severe asthma is defined as asthma that remains uncontrolled despite appropriate treatment with high-dose inhaled corticosteroids (ICS) and a long-acting bronchodilator, ensuring proper treatment adherence, inhalation technique, and management of triggers and comorbidities. It is also considered severe when symptoms get worse or severe exacerbations occur after treatment reduction.^1^, ^2^, ^3^ Globally, severe asthma accounts for approximately 3.7–10 of all asthma cases, being T2 phenotype, both allergic and eosinophilic, the most common, showing a prevalence of 40–80.^1^^,^^2^^,^^4^^,^^5^

Since 2003, the first biologic therapy was approved as an additional option for the management of severe T2 phenotype asthma, demonstrating excellent outcomes.^1^^,^^3^^,^^4^ With the advent of precision medicine, prognostic factors and predictors of response have been better identified, facilitating the selection of the most appropriate biologic therapy for each patient based on their phenotype and endotype. Eosinophil count, fractional exhaled nitric oxide (FeNO), clinical characteristics, comorbidities, and safety are all considered.^3^ However, current consensus statements and clinical guidelines do not specify the exact duration for maintaining biologic therapy, creating uncertainty among treating physicians.

In current clinical practice, biologic therapy for severe asthma is often maintained indefinitely due to the lack of clear evidence. However, some studies have evaluated treatment discontinuation without finding an increased risk of exacerbations after withdrawal, while others report the opposite.^6^ Although there are clear recommendations on how to initiate and select biologic therapy, it remains necessary to determine whether it should be continued indefinitely or if it can be reduced and eventually withdrawn once asthma remission is achieved, similar to the approach used with other therapies.^1^^,^^2^ This scoping review aims to identify when it is safe to taper or discontinue biologic therapy without triggering exacerbations or loss of disease control, based on the available literature.

## Methods

We conducted a scoping review based on the methodological framework proposed by Arksey and O'Malley and refined by the Joanna Briggs Institute (JBI).^7^ To report this study, we followed the PRISMA Extension for Scoping Reviews (PRISMA-ScR).

### Objective and research question

The objective was to describe and summarize the available evidence on when and how it is safe to taper or discontinue biologic therapy in the management of severe asthma in remission, without causing exacerbations or loss of symptom control, according to recommendations and protocols described in the literature.

### Search strategy

The search strategy included a review of PubMed, EMBASE, Epistemonikos, and LILACS from their inception to May 27, 2024. The search utilized MeSH terms, keywords, free-text terms, and Boolean operators. The complete search strategy is presented in Supplementary Table 1.

### Eligibility criteria

The following inclusion criteria were applied: (1) full-text original or secondary articles in any language; and (2) studies addressing the tapering or discontinuation of biologic therapy in the management of patients with severe asthma in remission. Exclusion criteria included articles related to animal studies, in vitro research, study protocols, letters to the editor, conference abstracts, surveys, author commentaries, case reports, and case series.

### Selection of eligible studies

According to the eligibility criteria, 2494 articles were imported into Rayyan (https://www.rayyan.ai/), a web-based collaborative platform for systematic reviews. Duplicate records were identified and removed. Subsequently, 4 reviewers independently screened the titles and abstracts, applying the inclusion criteria using Rayyan's blinding feature. Any disagreements regarding inclusion were resolved through in-depth discussion until consensus was reached among the 4 reviewers.

### Data extraction

Data extraction was carried out independently by 4 reviewers using a standardized data extraction template in Microsoft Excel. Extracted information included study characteristics (author, year of publication, country, study design), biologic agent used, duration of treatment, outcomes following tapering or discontinuation of the biologic (symptom control, exacerbations, T2 inflammatory response, lung function, use of systemic steroids, maintenance of remission), follow-up period after discontinuation, and author recommendations. Any discrepancies that were found during the data extraction process were resolved by consensus among the 4 reviewers.

### Data synthesis and analysis

A narrative approach was used to synthesize and analyze the data. Based on the information gathered, key themes were proposed to facilitate a critical synthesis of the findings. Two synthesis tools were implemented: the first was a summary table, and the second was the presentation of the main themes in the results section.

## Results

### Literature search

**Fig. 1:**
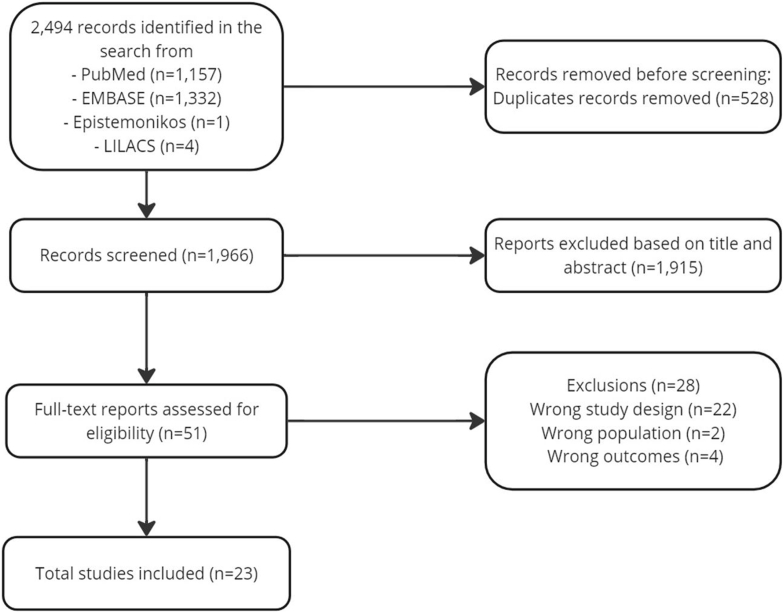
PRISMA flow diagram – Records selection.

The initial search yielded 2494 records, which were reduced to 1966 after the removal of 528 duplicates. Each record was screened based on the predefined eligibility criteria, identifying 51 potentially relevant articles for full-text review. Upon further examination, 28 were excluded due to study design (n = 19 conference abstracts, n = 1 author commentary, n = 1 research letter, n = 1 case series), inappropriate outcomes (n = 4 did not address biologic therapy discontinuation for clinical reasons), or incorrect population (n = 2 involved patients with uncontrolled asthma). The selection process resulted in the inclusion of 23 articles in the scoping review (Fig. 1).

### Overview of included studies

A total of 23 studies published between 2009 and 2024 met the inclusion criteria, including 6 literature reviews, 5 experimental studies, and 12 observational studies. The mean age of participants ranged from 33 to 64.5 years, with 3 studies including pediatric populations.

The most frequently evaluated biologic was omalizumab, with discontinuation documented in 12 studies (2 experimental and 10 observational). mepolizumab was analyzed in 2 experimental studies, and tezepelumab in 1. Additionally, 1 study employed an artificial intelligence–based prediction model to assess the discontinuation of 5 biologics: omalizumab, mepolizumab, reslizumab, benralizumab, and dupilumab.

The duration of treatment prior to discontinuation ranged from 4 to 60 months, with follow-up periods after discontinuation extending up to 10 years. The main characteristics of the studies are summarized in Table 1, while the findings of the experimental and observational studies are presented in Table 2, and the literature reviews in Table 3.

**Table 1:** Summary of key data from experimental and observational studies included in the scoping review.

Article	Year	Design	Sample Size	Age (years) mean (SD)	Biologic	Duration of treatment with biologic	Follow-up time after discontinuation of biologic
Biomarkers and clinical outcomes after tezepelumab cessation extended follow-up from the 2-year DESTINATION study^8^	2024	Clinical trial	289 tezepelumab 137 placebos	47.1 (17.8)	tezepelumab	2 years	40 weeks
Health outcomes after stopping long-term mepolizumab in severe eosinophilic asthma: COMET^16^	2022	Clinical trial	151 out of 295 discontinued the biologic	56.1 (11.46)	mepolizumab	3 years	52 weeks
Stopping versus continuing long-term mepolizumab treatment in severe eosinophilic asthma (COMET study)^9^	2021	Clinical trial	151 out of 295 discontinued the biologic	56.1 (11.46)	mepolizumab	3 years	52 weeks
A step-down protocol for omalizumab treatment in oral corticosteroid-dependent allergic asthma patients^20^	2018	Intervention study	35	52.5 (17)	omalizumab	1.5 years	6 months after each dose reduction
A randomized multicenter study evaluating Xolair persistence of response after long-term therapy^17^	2017	Clinical trial	88 out of 176 discontinued the biologic	51.9 (13.3)	omalizumab	1 years	52 weeks
A prediction model for asthma exacerbations after stopping asthma biologics^21^	2023	Predictive model development and validation study	3057	47.1 (17.1)	omalizumab, mepolizumab, reslizumab, benralizumab, dupilumab	Not reported	6 months
Asthma patients who stop asthma biologics have a similar risk of asthma exacerbations as those who continue asthma biologics^6^	2021	Retrospective cohort study	1247 out of 4958 discontinued the biologic	52.38 (17.37)	omalizumab, mepolizumab, benralizumab, reslizumab, dupilumab.	18 months	Between 6 and 12 months
The long-term effectiveness of omalizumab in adult patients with severe allergic asthma: Continuous Treatment Versus Boosting Treatment^18^	2021	Retrospective cohort study	14 out of 124 discontinued the biologic	60.8 (15.7)	omalizumab	4 months	12 months
Long-term effectiveness of omalizumab treatment in Thai severe asthmatic patients: A real-life experience^14^	2018	Cross-sectional study	22 out of 78 discontinued the biologic	58.5 (50.7–68.0)^a^	omalizumab	≥4 months	52 weeks
Real-life omalizumab exposure and discontinuation in a large nationwide population-based study of pediatric and adult asthma patients^12^	2022	Cross-sectional study	19,203	Adults 52.1 (15.6) Child 11.9 (3.2)	omalizumab	Between 49.3 and 56.4 months	10 years
Discontinuing omalizumab treatment in super-Responder patients with allergic severe asthma: Can the baseline total IgE level Be used as a Biological Marker to Decide discontinuing omalizumab treatment?^13^	2022	Cross-sectional study	14 out of100 super-responder patients discontinued the biologic	49.8 (13.1)	omalizumab	Minimum 6 months (average 5 years)	5 years
Small, prospective, observational, pilot study in patients with severe asthma after discontinuation of omalizumab treatment^10^	2018	Observational pilot study	12	40.6 (9.2)	omalizumab	29.5 months (range: 11–61 months)	12 months
Asthma symptom re-emergence after omalizumab withdrawal correlates well with increasing IgE and decreasing pharmacokinetic concentrations^22^	2009	Clinical trial	152 volunteers in the single-dose bioequivalence study and 476 patients in the INNOVATE multiple-dose study who had their therapy discontinued	42 (14)	omalizumab	28 weeks	16 weeks
Effectiveness of the polish program for the treatment of severe allergic asthma with omalizumab: a single-center experience^19^	2016	Prospective cohort study	14 out of 53 discontinued the biologic	45.8 (15.3)	omalizumab	≥36 months	Not reported
After 6 years with Xolair; a 3-year withdrawal follow-up^11^	2010	Cross-sectional study	18	50 (39–73)^a^	omalizumab	6 years	3 years
Observational study in severe asthmatic patients after discontinuation of omalizumab for good asthma control^15^	2014	Cross-sectional study	61	40.7 (20.31)	omalizumab	22.7 months	12.1 months
CD-sens and clinical changes during withdrawal of Xolair after 6 years of treatment^23^	2007	Prospective cohort study	18	48 (37–71)^a^	omalizumab	6 years	Between 12 and 14 months

a Median (Q1 – Q3).

**Table 2:** Asthma control after biologic discontinuation in experimental and observational studies included in the scoping review.

Article	Symptom control	Exacerbations	Type 2 inflammatory response	Lung function	Systemic steroid use	Conclusion
Biomarkers and clinical outcomes after tezepelumab cessation extended follow-up from the 2-year DESTINATION study^8^	ACQ-5 scores increased between weeks 10–16, reaching levels similar to placebo. 22 (62/282) maintained remission	32.9 (95/289) of those who discontinued tezepelumab experienced ≥1 exacerbation vs. 27 (37/137) in the placebo group. Time to first exacerbation: 29 vs. 30 weeks	Serum eosinophils and FeNO increased from week 4–10. Total IgE increased from week 28 onward without returning to baseline values	Pre-BD FEV1 showed a gradual but stable decline	Not reported	Tezepelumab discontinuation led to a progressive decline in clinical control and biomarker suppression. However, asthma control and inflammatory markers remained better than baseline, suggesting a prolonged yet transient effect, which could be relevant for patients needing treatment pauses
Health outcomes after stopping long-term mepolizumab in severe eosinophilic asthma: COMET^16^	Worsening in daily symptom scores. Higher likelihood of mild symptoms in those who continued mepolizumab (p < 0.001)	Increased use of rescue medication and greater need for unscheduled medical care	Rise in serum eosinophil counts to pre-treatment levels	Morning PEF declined from week 4 onward	Not reported	Mepolizumab discontinuation resulted in a loss of clinical benefits, with no evidence of sustained remission. Monitoring morning PEF may be key to detecting early deterioration after treatment withdrawal
Stopping versus continuing long-term mepolizumab treatment in severe eosinophilic asthma (COMET study)^9^	Decrease in asthma control (HR 1.52; 95 CI: 1.13–2.02; p = 0.005)	Higher risk of significant clinical exacerbation (HR 1.61; 95 CI: 1.17–2.22; p = 0.004). Severe exacerbations requiring hospitalization or emergency care were infrequent	Elevated serum eosinophil count at week 52 (270 vs. 40 cells/μL)	No significant difference in Pre-BD FEV1 between groups (p = 0.186)	Not reported	Mepolizumab discontinuation increased exacerbations and reduced asthma control, even after 3 years of use, suggesting a loss of clinical benefits and quality of life
A step-down protocol for omalizumab treatment in oral corticosteroid-dependent allergic asthma patients^20^	Not reported	No severe exacerbations requiring emergency care or hospitalization were reported	Greater reduction in eosinophils in the tolerant group; IgE increased in all groups; no significant differences in FeNO	In the tolerant group, FEV1 increased by 10.9 ± 21.4 in the first month and remained higher than in other groups during follow-up. The intolerant group had the worst outcomes, and partially tolerant group resembled it by month 48	In the tolerant group, oral corticosteroid use was reduced to zero in all cases	34.3 tolerated complete omalizumab withdrawal, while in 42.9 dose modification was not feasible. No definitive predictive phenotype was identified, although a 100 mL FEV1 improvement in the first month and a 50 reduction in oral corticosteroid use by month 6 may be success markers
A randomized multicenter study evaluating Xolair persistence of response after long-term therapy^17^	Patients who continued omalizumab showed better asthma control in both the asthma control test score and the asthma control questionnaire (p = 0.0188 and p = 0.0039, respectively)	67 of patients in the omalizumab group had no exacerbations vs. 47.7 in the placebo group (relative reduction of 40.1). Time to first exacerbation was significantly longer in the omalizumab group (HR 0.49; 95 CI 0.28–0.86)	Placebo group, free IgE levels increased and FcεRI (high-affinity IgE receptor) expression on basophils was significantly higher than in the omalizumab group (p < 0.0001). Peripheral eosinophils increased in the placebo group with exacerbations after withdrawal (p < 0.001)	No significant difference in FEV1 change between groups up to week 52	Not reported	Patients continue to benefit from long-term omalizumab. FeNO and blood eosinophils could be useful biomarkers to guide treatment decisions
A prediction model for asthma exacerbations after stopping asthma biologics^21^	Not reported	Failure probability after withdrawal was slightly higher in those who discontinued the biologic, but not statistically significant	Not reported	Not reported	Not reported	Fewer exacerbations and fewer outpatient visits in the previous 6 months increase the likelihood of successful discontinuation. Comorbidities such as chronic urticaria or atopic dermatitis were associated with better response after biologic withdrawal
Asthma patients who stop asthma biologics have a similar risk of asthma exacerbations as those who continue asthma biologics^6^	Not reported	Among all who discontinued the biologic, 127 failed withdrawal within 6 months (10.2), defined as a 50 or more increase in exacerbations	Not reported	Not reported	Not reported	Discontinuation of asthma biologics may not be associated with increased risk of asthma exacerbations. A rise in asthma exacerbations was infrequently observed among those who stopped treatment, with rates similar to those who continued using asthma biologics
The long-term effectiveness of omalizumab in adult patients with severe allergic asthma: Continuous Treatment Versus Boosting Treatment^18^	Significant improvements in ACT in the continuous treatment group, not in the reinforcement group	Significant increase in the reinforcement group (0.7 ± 1.4 vs. 2.9 ± 3.6, p = 0.031). Fewer exacerbations in the continuous treatment group (15 vs. 50)	Not reported	Not reported	Continuous treatment with omalizumab showed a significant reduction in the frequency of OCS use as controllers	The “reinforcement” strategy with omalizumab for 4 months was associated with worse clinical outcomes at 12 months, while continuous treatment showed clear benefits
Long-term effectiveness of omalizumab treatment in Thai severe asthmatic patients: A real-life experience^14^	27 of patients who discontinued omalizumab relapsed and needed to restart treatment	Relapse occurred on average 6.5 months after suspension and required systemic corticosteroids	Total serum IgE before restarting: 355.7 ± 130.2 IU/mL	Not reported	Not reported	The suspension of omalizumab was associated with relapse in a significant proportion of patients, highlighting the benefit of continuous treatment
Real-life omalizumab exposure and discontinuation in a large nationwide population-based study of paediatric and adult asthma patients^12^	In adults, 70 maintained control at 1 year, 39 at 2 years, 24 at 3 years. In children, 76 at 1 year, 44 at 2 years, 33 at 3 years	Not reported	Not reported	Not reported	OCS use increased after suspension: 20 before vs. 33.3 in adults and 24.6 in children at 2 years	Many patients maintained asthma control after discontinuing omalizumab, although with an increase in OCS use, in a study with over 10 years of follow-up
Discontinuing omalizumab treatment in super-Responder patients with allergic severe asthma: Can the baseline total IgE level Be used as a Biological Marker to Decide discontinuing omalizumab treatment?^13^	36 of patients restarted omalizumab after a mean of 15.6 months due to worsening	Not reported	There were no significant differences in eosinophils or total IgE between those who restarted treatment and those who did not	There were no significant differences in FEV1 between groups	Not reported	The immunomodulatory effect of omalizumab may persist after discontinuation. Higher baseline total IgE levels may predict relapse, but further studies are needed
Small, prospective, observational, pilot study in patients with severe asthma after discontinuation of omalizumab treatment^10^	The ACT score remained stable 12 months after treatment discontinuation (p = 0.002). No patient reported a decrease in symptom scores	There were 2 severe exacerbations (1 in each patient) at 6 and 12 months after discontinuation, respectively	Significant decrease in SPT to the primary allergen at 6 months; total IgE increased during treatment and returned to baseline levels 6 months after stopping omalizumab	No significant worsening of lung function	Not reported	Patients good responders, non-smokers, and without concurrent pulmonary conditions maintained clinical stability after discontinuation, suggesting a sustained benefit of omalizumab
Asthma symptom re-emergence after omalizumab withdrawal correlates well with increasing IgE and decreasing pharmacokinetic concentrations^22^	Correlates with free IgE levels and pharmacokinetics	Not reported	Free IgE is rapidly suppressed after initiating omalizumab, with no differences between responders and non-responders	Changes in lung function show hysteresis relative To IgE	Not reported	Dose reduction of omalizumab is not recommended, as increased free IgE impairs asthma control
Effectiveness of the polish program for the treatment of severe allergic asthma with omalizumab: a single-center experience.^19^	Not reported	After discontinuing omalizumab, 11 out of 14 (79) patients experienced worsening asthma control and severe exacerbations	Not reported	Not reported	Not reported	Following the discontinuation of the omalizumab program, frequent severe exacerbations were observed, mainly in patients whose asthma had not been previously controlled with high doses of OCS
After 6 years with Xolair; a 3-year withdrawal follow-up^11^	12 out of 18 patients showed no change or improvement after 3 years without omalizumab	16 out of 18 patients did not report increased nocturnal attacks; 14 out of 18 did not increase their medication	Significant decrease in basophil allergen sensitivity (CD-sens) to cat and dust mite allergens. Patients with poorly controlled asthma showed higher CD-sens	Not reported	Not reported	After 3 years without omalizumab, most patients had mild and stable asthma, possibly due to reduced CD-sens sensitivity
Observational study in severe asthmatic patients after discontinuation of omalizumab for good asthma control^15^	55.7 of patients lost asthma control after an average of 13 months; those with an “excellent” response maintained control for a longer time	96.7 of patients had no exacerbations before discontinuation	Not directly reported.	Not directly reported.	82 of cases did not require oral corticosteroids before discontinuation	Asthma control was maintained in approximately half of the patients after discontinuation, especially in those with long-term treatment. Further studies are needed to define discontinuation criteria
CD-sens and clinical changes during withdrawal of Xolair after 6 years of treatment^23^	Most patients had mild asthma after 12–14 months without treatment	Not reported	Downregulation of basophil reactivity and increased allergen-specific IgG4 to cat allergens	No significant changes in FEV1 or PEF after discontinuation	Not reported	After 12–14 months without omalizumab, most patients maintained mild and stable asthma, with unchanged lung function

ACQ-5 Asthma Control Questionnaire 5. FeNO Fractional exhaled nitric oxide.

FEV1 Forced expiratory volume in 1 s. OCS Oral corticosteroids. CD-sens Basophil sensitivity. PEF Peak expiratory flow.

**Table 3:** Conclusions from literature reviews included in the scoping review.

Article	Year	Design	Biologic	Conclusions
Long-term safety, durability of response, cessation and switching of biologics^27^	2024	Literature review	omalizumab, mepolizumab, benralizumab, reslizumab, dupilumab, tezepelumab	The possible factors for discontinuing a biologic include: 1) asthma remission induced by treatment, 2) low levels of T2 inflammation biomarkers before starting the biologic, 3) absence of T2 inflammation due to previous comorbidities, 4) no dependence on OCS, 5) low frequency of previous exacerbations, and 6) use of low ICS-LABA doses before starting the biologic
Feasibility of discontinuing biologics in severe asthma: An Algorithmic Approach^28^	2021	Literature review	omalizumab, mepolizumab, reslizumab, benralizumab and dupilumab	Super-responders could discontinue the biologic if they meet all criteria: Absence of symptoms (ACQ <1.5 or ACT >19), no exacerbations or use of corticosteroids, normal lung function (FEV1 ≥80), suppressed T2 inflammation (eosinophils <300 μL and FeNO <50 ppb), and controlled allergic comorbidities
Biological therapy management from the initial selection of biologics to switching between biologics in severe asthma^30^	2023	Literature review	omalizumab, mepolizumab, reslizumab, benralizumab or dupilumab	Discontinuation of biologics could be considered in severe asthma patients who have no symptoms, exacerbations in the last year, or need for oral corticosteroids, with suppressed T2 inflammation (eosinophils and FeNO) and controlled allergic comorbidities. A minimum use of 5 years is recommended to avoid loss of control
Omalizumab for severe allergic asthma: 7 Years and open questions^24^	2014	Literature review	omalizumab	PK/PD models suggest that IgE synthesis rate is reduced with omalizumab, which could allow for dose reduction or discontinuation after 6 years. If treatment is resumed after >12 months, it is recommended to measure total Ig E again for dose adjustment
Concept of remission in severe asthma^25^	2024	Literature review	omalizumab, mepolizumab, reslizumab, benralizumab, dupilumab or tezepelumab	In patients with prolonged remission, it is suggested to adjust the biologic dose starting with the standard dose and gradually reducing it, either by spacing out applications or reducing the dose, with monitoring every 3–6 months. If asthma remains controlled, reductions continue; if it worsens, the dose is increased. The goal is to find the minimal effective dose to optimize treatment and reduce side effects
Biologics for severe asthma: The real-world evidence, effectiveness of switching, and prediction factors for the efficacy.^29^	2023	Literature review	omalizumab, mepolizumab, reslizumab, benralizumab or dupilumab	Based on previous studies and consensus definitions, criteria for discontinuing biologics suggest similar guidelines to other reviews: Complete clinical control, normalized lung function, suppressed T2 inflammation, and controlled comorbidities

OCS Oral corticosteroids. ACQ-5 Asthma Control Questionnaire 5. ACT Asthma Control Test. FEV1 Forced expiratory volume in 1 s. PK/PD Pharmacokinetic/pharmacodynamic model. FeNO Fractional exhaled nitric oxide.

### Asthma control following discontinuation of biologic therapies

Among patients treated with tezepelumab, 33.5 achieved complete clinical remission after 2 years of therapy, of whom 22 (62/282) maintained this status between weeks 104 and 140 following discontinuation. However, between weeks 10 and 16 post-discontinuation, a decline in Asthma Control Questionnaire (ACQ-6) scores was observed.^8^

In the COMET study, upon discontinuation of mepolizumab, 11 of patients continued to require prednisone (5 mg/day), and 5 had experienced severe exacerbations in the year prior to withdrawal. At 52 weeks of follow-up, the difference in ACQ-5 scores between those who continued versus those who discontinued the biologic was 0.23 (−0.02–0.48; p = 0.067). In the discontinuation group, asthma remained partially controlled (ACQ-5: 1.2 ± 1.04), with FEV_1_ at 65.5 ± 19.6 and eosinophil counts within normal ranges. The study concluded that in some patients, discontinuation of mepolizumab may not be associated with a significant loss of asthma control, although a subset may require closer monitoring to prevent relapse.^9^

Krcmová et al evaluated the impact of omalizumab discontinuation in 12 patients with prior clinical stability, it means, Asthma Control Test (ACT) ≥20 and no severe exacerbations after at least 16 weeks of treatment. At 12-month follow-up, ACT scores remained stable (p = 0.002), with a mean treatment duration of 29.5 months (range: 11–61). Three years after discontinuing a 6-year treatment, 44.4 (8/18) reported less severe symptoms compared to the period during which they received the biologic. These findings suggest that discontinuation of omalizumab may not lead to an immediate loss of asthma control, and in some cases, symptoms may even improve over the long term.^10^

Nopp et al found that 3 years after omalizumab discontinuation, 12 out of 18 patients reported improvement or stability in asthma control compared to the period of continuous treatment. Most patients remained clinically stable. However, 2 out of 18 experienced an increase in nocturnal attacks, and 14 out of 18 showed minimal or no increase in medication requirements. In the long term, a significant proportion of patients maintained mild and stable asthma following biologic withdrawal.^11^

In adults treated with omalizumab for an average of 4 years (95 CI: 49.3–53.4 months), 70, 39, and 24 remained controlled without resuming the biologic at 1, 2, and 3 years, respectively. In children, with a mean treatment duration of 53.7 months (95 CI: 50.6–56.4), these rates were 76, 44, and 33.^12^ On the other hand, Arslan B et al found that approximately one-third of patients with controlled asthma after 5 years of treatment required restarting omalizumab within 3 years of discontinuation.^13^ Kawamatawong et al reported that 27 of patients resumed the biologic due to symptom relapse, with a shorter mean duration of treatment prior to discontinuation (9.7 ± 6.3 months).^14^

Molimard et al identified a loss of asthma control in 55.7 of patients after discontinuing omalizumab, with a mean time to relapse of 13 months (mean 20.4 ± 2.6; 95 CI: 8.3–28.1). Among these patients, 69.2 had received treatment for less than 1 year, 59.1 for 1 to 2 years, and 46.1 for more than 2 years. However, no correlation was found between treatment duration or dosage. Upon biologic reinitiation, the response was excellent in 52.5 of patients (control maintained for 17 months) and good in 47.5 (control for 12.8 months). In the same study, discontinuation in patients treated for more than 3.5 years was not associated with early loss of control (<6 months post-discontinuation). Of the 34 patients who relapsed, 20 (58.8) resumed omalizumab, with an excellent response in 30, good in 40, no response in 20, and not assessed in 10.^15^

### Impact of biologic discontinuation on the frequency and severity of asthma exacerbations

Brightling et al reported that following the discontinuation of tezepelumab, 32.9 (95/289) of patients experienced at least 1 exacerbation within 40 weeks after the last dose, compared to 27 (37/137) in the placebo group. The time to first exacerbation was similar in both groups: 29 weeks in patients previously treated with tezepelumab and 30 weeks in the placebo group.^8^

In the case of mepolizumab, 59 of patients who discontinued treatment experienced at least 1 clinically significant exacerbation within the year following discontinuation, compared to 46 of those who continued treatment. Additionally, the time to the first exacerbation was significantly shorter in the discontinuation group.^9^ Liu et al found that, at 52 weeks after stopping mepolizumab following 3 years of treatment, 26.1 of patients experienced asthma worsening, compared to 16.2 of those who remained on the biologic. Patients who discontinued treatment showed higher use of rescue medication (HR 1.36; 95 CI: 1.00–1.84; p = 0.047) and were more likely to require unscheduled healthcare resources (HR 1.81; 95 CI: 1.31–2.49; p < 0.001).^16^

Regarding omalizumab, Krcmová et al found that 67 of patients who continued treatment experienced no exacerbations, compared to 47.7 of those who discontinued it, with an absolute difference of 19.3 (95 CI: 5.0–33.6). Moreover, the time to first exacerbation was significantly longer in the group that maintained treatment (HR 0.49; 95 CI: 0.28–0.86).^17^

Huang et al analyzed the frequency of exacerbations in patients who discontinued omalizumab after 4 months of treatment, observing a significant increase in the number of exacerbations during the 12 months following discontinuation compared to the previous year (baseline = 0.7 ± 1.4; follow-up = 2.9 ± 3.6; p = 0.031). In contrast, 15 of patients who continued omalizumab experienced exacerbations, compared to 50 of those who discontinued it.^18^

Humbert et al evaluated adult and pediatric patients with controlled asthma who discontinued omalizumab after at least 16 weeks of treatment. After a median treatment duration of 51.2 months in adults (95 CI: 49.3–53.4) and 53.7 months in children (95 CI: 50.6–56.4), healthcare resource utilization, including asthma-related hospitalizations, remained stable 2 years after discontinuation: none before discontinuation, and 1.3 in adults and 0.6 in children 2 years post-discontinuation.^12^

Kupryś-Lipińska et al reported that following the discontinuation of omalizumab after ≥36 months of treatment, 79 (11/14) of patients experienced a deterioration in asthma control and severe exacerbations, particularly among those receiving high doses of oral corticosteroids.^19^

In the OMADORE study, Domingo et al reported that 34.3 of patients tolerated a gradual dose-reduction protocol of omalizumab leading to complete discontinuation, while 22.9 tolerated dose reduction but not full withdrawal. In the group who successfully discontinued the biologic, oral corticosteroid use was reduced to zero by the end of follow-up. In the partially tolerant group, oral corticosteroid use showed a downward trend, whereas no relevant changes were observed in the intolerant group.^20^

In contrast, Jeffery et al, in an analysis of a database comprising 4960 patients treated with biologics for asthma, found that 10.2 of the 1249 patients who discontinued treatment after 6–12 months experienced a ≥50 increase in exacerbation rates in the following 6 months, compared to 9.5 of the 1247 patients who continued treatment for at least 18 months. No significant increase in exacerbation risk was associated with biologic discontinuation, with a similar adjusted odds of treatment failure between the groups (OR 1.085; 95 CI: 0.833–1.413).^6^

Finally, Inselman et al developed and validated a predictive model to evaluate the risk of exacerbations following biologic discontinuation in 3057 patients. They identified that 552 patients (22.6) experienced an exacerbation within 6 months of treatment cessation. The study concluded that fewer prior exacerbations, fewer outpatient visits, and fewer treatment days in the 6 months preceding discontinuation, along with the presence of chronic spontaneous urticaria or atopic dermatitis, and the absence of sinusitis or COPD, were associated with a successful biologic withdrawal.^21^

### Impact of biologic discontinuation on type 2 inflammatory response

In the study by Brightling et al, following the last dose of tezepelumab, a gradual increase in blood eosinophil counts and FeNO levels was observed between weeks 4 and 10. However, these values did not return to baseline levels.^8^ In contrast, patients who discontinued long-term treatment (≥3 years) with mepolizumab experienced an increase in serum eosinophil levels that returned to pre-treatment values.^9^

In the study by Ledford et al, the discontinuation of malizumab was assessed in patients who had been treated for at least 1 year and had no exacerbations in the 2 months prior to withdrawal. At baseline, there were no significant differences in eosinophil counts between those who continued versus discontinued treatment (p = 0.70), and this trend remained through week 52 (p = 0.76). However, among those who discontinued, patients who experienced exacerbations showed a significantly difference in the eosinophil counts compared to those without exacerbations (p < 0.001). Additionally, an increase in FeNO levels was observed from baseline to week 12, which correlated with a higher risk of exacerbations (p = 0.038).^17^

Slavin et al, using a pharmacokinetic/pharmacodynamic (PK/PD) model, demonstrated that free IgE levels are rapidly suppressed upon initiation of omalizumab, in both responders and non-responders. This suppression occurs before improvements are observed in total symptom scores, peak expiratory flow, or rescue medication use. Upon treatment discontinuation, free IgE levels returned to baseline between weeks 18 and 20, which likely contributed to symptom recurrence.^22^

On the other hand, Krcmová et al observed that following omalizumab discontinuation, there was no significant increase in absolute eosinophil counts (p = 0.337), and there were not relevant changes in FeNO levels, with skin prick test reactivity to the primary allergen remaining low.^10^ Nopp et al. also found that IgE levels did not differ before, during, or after treatment. Moreover, between 12 and 14 months after completing a six-year treatment, most patients presented with mild asthma, stable lung function, and reduced basophil reactivity.^23^

Three years after treatment cessation, basophil sensitivity (CD-sens) to cat and mite allergens remained significantly lower compared to controls (p < 0.02), although it was higher in patients with partially controlled or uncontrolled asthma, with no changes observed in IgE levels.^11^ Finally, out of 14 patients who responded to omalizumab, 5 (36) restarted treatment after an average of 15.6 ± 12.4 months. No significant differences were found in eosinophil counts, total IgE levels, or FEV1 between those who resumed treatment and those who did not, both at baseline and at five-year follow-up.^13^

### Impact of biologic discontinuation on lung function

Following tezepelumab discontinuation, a progressive decline in FEV_1_ was observed between weeks 10 and 16, reaching levels comparable to those of the placebo group.^8^ Similarly, discontinuation of mepolizumab led to a deterioration in morning peak expiratory flow (PEF) starting at week 4 (HR 1.77; 95 CI: 1.21–2.59; p = 0.003).^16^

In the case of omalizumab, Ledford et al found no significant differences in FEV_1_ between patients who continued or discontinued treatment over 52 weeks.^17^ Similarly, Krcmová et al reported no relevant changes in lung function following biologic discontinuation in patients without pulmonary comorbidities, although 2 patients experienced severe exacerbations at 6 and 12 months.^10^ Nopp et al confirmed clinical and functional stability after treatment withdrawal for over a year, with most patients reporting asthma symptoms that were similar or even milder, and no increase in nocturnal exacerbations.^23^

The study by Solèr indicated that gradual withdrawal of omalizumab was associated with better long-term spirometric values compared to abrupt discontinuation. Moreover, his theoretical analysis suggests that prolonged treatment reduces IgE production by 50 per year, which could allow for dose adjustment and consideration of treatment cessation after 6 years.^24^ Cohn proposed a stepwise approach to biologic dose reduction in asthma, with monitoring every 3–6 months and adjustments based on patient response, aiming to optimize control, minimize adverse effects, and reduce costs.^25^^,^^26^

## Discussion

The reviewed studies highlight the lack of consensus regarding the optimal timing for discontinuing biologics in patients with moderate to severe asthma who have shown a good response to treatment. Mohan et al proposed criteria for assessing the feasibility of treatment interruption, emphasizing asthma remission, absence of significant type 2 inflammation, no use of oral corticosteroids (OCS), and a low frequency of prior exacerbations.^27^ Similarly, Hamada et al and Nagase et al identified “super-responders” as ideal candidates for treatment discontinuation, characterized by the absence of symptoms and exacerbations, stable lung function, and low inflammatory biomarkers.^28^^,^^29^ Yilmaz et al support this approach, suggesting a minimum of 5 years of treatment to reduce the risk of early relapse.^30^

The duration of biologic treatment prior to discontinuation ranged from 4 months to 6 years, with follow-up periods varying from 4 months to 10 years. In the case of tezepelumab, a progressive loss of clinical asthma control and type 2 inflammatory biomarkers was observed following treatment cessation, although the leves did not returned to baseline.^8^ Similarly, the discontinuation of mepolizumab was associated with decreased asthma control, suggesting the need for continuous use.^9^^,^^16^ However, 1 observational study involving various biologics found no increased risk of exacerbations after discontinuation.^6^

Regarding omalizumab, the evidence shows variable outcomes. Three studies reported that discontinuation after 4–7 months led to a rapid increase in free IgE and a rise in exacerbations.^14^^,^^18^^,^^22^ However, discontinuation after 1 year resulted in a gradual decline in symptom control without significant differences in exacerbation rates or lung function.^17^

The OMADORE study implemented a gradual dose reduction protocol for omalizumab prior to discontinuation, yielding positive outcomes in patients with well-controlled asthma and no need for oral corticosteroids (OCS): 34.3 of patients tolerated treatment withdrawal, and 22.9 were able to reduce the dose without relapse.^20^ Similarly, Molimard et al found that after 22.7 months of treatment, asthma control was maintained in half of the patients following discontinuation, particularly in those with prolonged biologic use.^15^ Krcmová et al reported clinical stability 12 months after discontinuation in omalizumab responders without pulmonary comorbidities or smoking history.^10^ However, Kupryś-Lipińska et al documented severe exacerbations in patients with uncontrolled disease and high OCS use after treatment cessation.^19^

The study by Arslan et al suggests a possible prolonged immunomodulatory effect in patients treated with omalizumab for 5 years, of whom only one-third required retreatment within 3 years after discontinuation.^13^ Furthermore, studies in patients treated for up to 6 years indicate that most maintained mild and stable asthma following discontinuation, with no deterioration in lung function.^11^^,^^23^ Humbert et al reported that 10 years after stopping omalizumab following a four-year treatment period, patients continued to demonstrate low healthcare resource utilization.^12^

One limitation of this analysis is the predominance of studies focused on omalizumab, with limited evidence available for other biologics. Additionally, the heterogeneity in study designs and outcomes makes direct comparison of results challenging. Only 1 study assessed a structured dose-reduction protocol, and in many studies, the success of discontinuation was evaluated solely based on exacerbation rates, without considering symptom control or inflammatory biomarkers.

As a strength, this analysis provides a comprehensive and reproducible review of biologic discontinuation in controlled asthma, particularly with omalizumab. The findings offer key insights into potential candidates for therapy withdrawal and the short-, medium-, and long-term outcomes.

Based on the findings of the present study, we consider it clinically relevant for future research to explore which criteria or biomarkers—considering individual patient characteristics—may predict a sustained response to biologic therapy in patients with severe asthma following treatment discontinuation.

## Conclusion

The reviewed evidence suggests that discontinuation of biologics in moderate to severe asthma should be individualized. A minimum treatment period of 5 years may be appropriate before considering withdrawal in patients with sustained control, stable lung function, and suppressed inflammatory biomarkers. Discontinuation of tezepelumab and mepolizumab is associated with a progressive decline in asthma control, whereas omalizumab withdrawal shows heterogeneous outcomes depending on treatment duration and clinical response. Gradual dose-reduction strategies may help optimize treatment withdrawal, minimizing relapses and costs without compromising disease control. Ultimately, decision-making should be based on a personalized assessment, considering clinical evolution, inflammatory biomarkers, and individual patient characteristics.

## Abbreviations

ICS, nhaled corticosteroids; FeNO, Fractional exhaled nitric oxide; JBI, Joanna Briggs Institute; PRISMA-ScR, PRISMA Extension for Scoping Reviews; ACQ-5, Asthma Control Questionnaire 5; ACT, Asthma Control Test; FEV1, Forced expiratory volume in 1s; OCS, Oral corticosteroids; PK/PD, Pharmacokinetic/pharmacodynamic model; CD-sens, Basophil sensitivity; PEF, Peak expiratory flow.

## Data availability statement

The data related to our study and materials will be available upon request by contacting the correspondence author (Liliana Fernández-Trujillo, liliana.fernandez@fvl.org.co).

## Authors’ contributions

All authors have read and approved the manuscript and significantly contributed to this paper. **JRV:** Conception and design, literature review, manuscript writing correction, and final approval of the manuscript. **CDIE:** Literature review, manuscript writing correction, and final approval of the manuscript. **JSGS:** Literature review, manuscript writing, correction, and final approval of the manuscript. **CSR:** Literature review, manuscript writing correction, and final approval of the manuscript. **LFT:** Conception and design, literature review, manuscript writing correction, and final approval of the manuscript.

## Ethics statement

The study was considered exempt from ethics because it was a review of the literature and did not involve human subjects.

## Authors’ consent for publication

All authors gave their consent for the publication of this manuscript.

## Declaration of Generative AI and AI-assisted technologies in the writing process

Nothing to disclose.

## Funding

No funding sources were used. This research did not receive any specific grants from funding agencies in the public, commercial, or not-for-profit sectors.

## Declaration of competing interest

The authors declare that they have no competing interests. All other authors have no conflict of interest within the scope of the submitted work. This manuscript has not been published and is not under consideration for publication elsewhere. Additionally, all of the authors have approved the contents of this paper and have agreed to the journal's submission policies.
