# Supplementary material for: Discontinuation of biologic therapy in severe asthma: Evidence and strategies for safe withdrawal: A scoping review
Source: World Allergy Organ J. 2025 Aug 22;18(9):101107. doi: 10.1016/j.waojou.2025.101107 (PMC12397818; doi:10.1016/j.waojou.2025.101107)
Supplement: Multimedia component 1 [file mmc1.docx]

**Supplementary Table 1.** Search terms used according to the electronic databases consulted [date: 27.05.2024]

| **Database** | **Search Strategy** | **Results** |
| --- | --- | --- |
| PudMed | ((Asthma[MeSH Terms]) AND ((((Biological therapy[MeSH Terms]) OR (Antibodies, Monoclonal[MeSH Terms])) OR (Omalizumab[MeSH Terms])) OR (Anti-IgE antibodies[MeSH Terms]))) AND (((((Stopping) OR (Completion)) OR (Discontinue therapy)) OR (Step-down treatment)) OR (Term therapy)) Filters: Humans | 1,157 |
|  |  |  |
| EMBASE | '('asthma'/de OR (((((((((((('asthma'/exp OR asthma OR uncontrolled) AND ('asthma'/exp OR asthma) OR 'difficult to treat') AND ('asthma'/exp OR asthma) OR severe) AND ('asthma'/exp OR asthma) OR 'moderate to') AND severe AND ('asthma'/exp OR asthma) OR uncontrolled) AND 'moderate to' AND severe AND ('asthma'/exp OR asthma) OR uncontrolled) AND severe AND ('asthma'/exp OR asthma) OR allergic) AND ('asthma'/exp OR asthma) OR severe) AND allergic AND ('asthma'/exp OR asthma) OR eosinophilic) AND ('asthma'/exp OR asthma) OR 't2'/exp OR t2) AND ('asthma'/exp OR asthma) OR 't2 high') AND ('asthma'/exp OR asthma))) AND ('biological'/de AND 'therapy'/de OR (monocolnal AND 'antibody'/de) OR 'omalizumab'/de OR 'dupilumab'/de OR 'mepolizumab'/de OR 'benralizumab'/de OR 'tezepelumab'/de OR ('immunoglobulin'/de AND e AND 'antibody'/de) OR (((((((((('biological'/exp OR biological) AND ('therapy'/exp OR therapy) OR monoclonal) AND ('antibodies'/exp OR antibodies) OR 'omalizumab'/exp OR omalizumab OR 'dupilumab'/exp OR dupilumab OR 'mepolizumab'/exp OR mepolizumab OR 'benralizumab'/exp OR benralizumab OR 'reslizumab'/exp OR reslizumab OR 'tezepelumab'/exp OR tezepelumab OR 'anti inmunoglobulin') AND e OR 'anti ige'/exp OR 'anti ige' OR anti) AND ('interleukin'/exp OR interleukin) AND 5 OR anti) AND ('il 5'/exp OR 'il 5') OR anti) AND ('interleukin'/exp OR interleukin) AND 5r OR anti) AND ('il 4'/exp OR 'il 4') AND il AND -13 OR 'anti interleukin 4rα' OR anti) AND thymic AND stromal AND ('lymphopoietin'/exp OR lymphopoietin) OR anti-) AND ('tslp'/exp OR tslp))) AND (((stopping OR 'completion'/exp OR completion OR discontinue) AND ('therapy'/exp OR therapy) OR 'step down') AND ('treatment'/exp OR treatment) OR term) AND ('therapy'/exp OR therapy) | 1,332 |
| Epistemonikos | Asthma OR Uncontrolled asthma OR Difficult-to-treat asthma OR Severe asthma OR Moderate-to- severe asthma OR Uncontrolled moderate-to- severe asthma OR Uncontrolled severe asthma OR Allergic asthma OR Severe allergic asthma OR Eosinophilic asthma OR T2 asthma OR T2-High asthma AND Biological therapy OR Monoclonal antibodies OR Omalizumab OR Dupilumab OR Mepolizumab OR Benralizumab OR Reslizumab OR Tezepelumab OR Anti-inmunoglobulin E OR Anti-IgE OR Anti interleukin 5 OR Anti Il-5 OR Anti interleukin 5R OR Anti IL-4IL -13 OR Anti-interleukin-4Rα OR Anti thymic stromal lymphopoietin OR Anti- TSLP AND Stopping OR Completion OR Discontinue therapy OR Step-down treatment OR Term therapy | 1 |
| LILACS | (((((((((((((Asthma) OR (Uncontrolled asthma)) OR (Difficult-to-treat asthma)) OR (Severe asthma)) OR (Moderate-to- severe asthma)) OR (Uncontrolled moderate-to- severe asthma)) OR (Uncontrolled severe asthma)) OR (Allergic asthma)) OR (Severe allergic asthma)) OR (Eosinophilic asthma)) OR (T2 asthma)) OR (T2-High asthma)) AND (((((((((((((((((Biological therapy) OR (Monoclonal antibodies)) OR (Omalizumab)) OR (Dupilumab)) OR (Mepolizumab)) OR (Benralizumab)) OR (Reslizumab)) OR (Tezepelumab)) OR (Anti-inmunoglobulin E)) OR (Anti-IgE)) OR (Anti interleukin 5)) OR (Anti Il-5)) OR (Anti interleukin 5R)) OR (Anti IL-4/IL -13)) OR (Anti-interleukin-4Rα)) OR (Anti thymic stromal lymphopoietin)) OR (Anti- TSLP))) AND (((((Stopping) OR (Completion)) OR (Discontinue therapy)) OR (Step-down treatment)) OR (Term therapy)) | 4 |
